# Nitinol Prosthesis in Stapes Surgery: Evolution from Heat-Activated to Superelastic Nitinol: A Systematic Review

**DOI:** 10.3390/jcm14041069

**Published:** 2025-02-07

**Authors:** Andrea Achena, Ludovica Pacelli, Carmine Prizio, Gabriella Mantini, Angelo Placentino, Remo Accorona, Valerio Valenzise, Francesco Pilolli, Giorgio Luigi Ormellese, Niccolò Mevio, Alberto Dragonetti

**Affiliations:** 1 Unit of Otorhinolaryngology, ASST Grande Ospedale Metropolitano Niguarda, 20162 Milan, Italy; ludovica.pacelli@unimi.it (L.P.); carmine.prizio@gmail.com (C.P.); gabriella.mantini@ospedaleniguarda.it (G.M.); remo.accorona@ospedaleniguarda.it (R.A.); valerio.valenzise@ospedaleniguarda.it (V.V.); francesco.pilolli@ospedaleniguarda.it (F.P.); giorgio.ormellese@ospedaleniguarda.it (G.L.O.); niccolo.mevio@ospedaleniguarda.it (N.M.); alberto.dragonetti@ospedaleniguarda.it (A.D.); 2 Department of Clinical Sciences and Community Health, University of Milan, 20090 Milan, Italy; 3 Division of Otolaryngology, Department of Surgical Sciences, University of Turin, 10126 Turin, Italy; 4 Division of Otorhinolaryngology, Garbagnate Milanese Hospital, Garbagnate Milanese, 20024 Milan, Italy; aplacentino@asst-rhodense.it

**Keywords:** otosclerosis surgery, stapes prosthesis, nitinol prostheses, air–bone gap reduction, hearing restoration, superelastic prostheses, heat-activated prostheses, complication rates in otosclerosis surgery, audiological outcomes, middle ear, artificial intelligence

## Abstract

**Background/Objectives:** Stapes surgery is a well-established treatment for conductive hearing loss caused by otosclerosis, with the choice of prosthesis playing a pivotal role in audiological outcomes and safety. Heat-activated and superelastic nitinol prostheses are widely used, but their comparative effectiveness and complication profiles remain debated. This systematic review and meta-analysis aimed to evaluate the audiological outcomes, complication rates, and overall performance of these two prosthesis types. **Methods:** A systematic review was conducted following PRISMA guidelines. Seven studies involving 273 patients were included. Data on mean air–bone gap (ABG) reduction, complication rates, and follow-up outcomes were extracted. Meta-analyses were performed using a random-effects model, and odds ratios (OR) with 95% confidence intervals (CI) were calculated for comparative analysis. **Results:** The pooled mean ABG reduction was 20.2 dB (95% CI: 19.47–20.95), indicating substantial and comparable improvements in hearing for both prosthesis types. Heat-activated prostheses achieved slightly higher ABG reduction in individual studies, while superelastic prostheses offered advantages in procedural simplicity. Complication rates were 6.0% for heat-activated and 5.6% for superelastic prostheses. The most common complications included sensorineural hearing loss (2.0–2.4%) and incus-related issues such as necrosis and lateralization. **Conclusions:** Both prosthesis types provide comparable audiological outcomes and surgical success rates. However, the slightly lower complication rate observed with superelastic prostheses emphasizes their safety and predictability. The choice of prosthesis should be guided by patient anatomy, surgeon expertise, and procedural considerations. Future studies should prioritize long-term outcomes and standardized reporting to further refine prosthesis selection.

## 1. Introduction

Otosclerosis is a degenerative condition of the middle ear that progressively fixates the stapes, leading to conductive hearing loss. Surgical treatment, typically through stapedotomy or stapedectomy, aims to restore sound transmission by replacing the immobilized stapes with a prosthesis. Over decades, advancements in prosthesis design and materials have significantly improved audiological outcomes while reducing postoperative complications. Early stapes prostheses made of materials such as stainless steel, platinum, and Teflon, while effective, often faced limitations in biocompatibility and long-term stability, leading to issues like prosthesis dislocation or extrusion [1].

The late 20th century marked a turning point in stapes surgery with the introduction of nitinol, an alloy of nickel and titanium. Nitinol’s shape memory and superelastic properties make it uniquely suited for middle ear surgery. The shape memory effect allows prostheses to “remember” a predetermined shape when exposed to specific temperatures, a feature exploited in heat-activated prostheses. Conversely, superelastic nitinol provides flexibility, enabling the prosthesis to adapt dynamically to anatomical structures without permanent deformation. One key feature is the self-crimping hook design, which adapts to the incus with gentle pressure (approximately 4–5 g), reducing the risk of incus necrosis, a common concern with excessive force during crimping [2]. These innovations have reduced the risk of dislocation and enhanced long-term stability [3,4].

Currently, heat-activated and superelastic nitinol prostheses are the two primary nitinol-based stapes prostheses. Heat-activated prostheses rely on thermal activation to achieve auto-crimping, securing their position around the incus with precision and minimizing manual manipulation by the surgeon. This reduces surgical time and potentially lowers the risk of displacement [5]. Superelastic nitinol prostheses, on the other hand, utilize their elastic deformation capabilities to adapt correctly to the thickness of the long process of the incus, offering superior performance in revision surgeries and reducing mechanical stress on surrounding tissues [6,7]. Leading manufacturers such as Kurz, Grace Medical, Audio Technologies, and Olympus have developed diverse designs tailored to patient-specific needs, incorporating features like self-crimping mechanisms and customizable sizes for improved surgical outcomes [8,9].

Despite these advances, the distinction between heat-activated and superelastic nitinol prostheses remains unclear in clinical practice. Both have shown excellent audiological outcomes, yet differences in complication rates, stability, and surgical applicability highlight the need for a comprehensive comparative analysis. This systematic review and meta-analysis aims to clarify these distinctions by evaluating the re-audiological performance, complication profiles, and technological advancements of heat-activated and superelastic nitinol prostheses. By consolidating the existing evidence, this study seeks to guide clinicians in selecting the most appropriate prosthesis for optimal patient outcomes in otosclerosis surgery.

## 2. Materials and Methods

The research and selection of articles were conducted with the assistance of artificial intelligence (AI) tools, facilitating a systematic and comprehensive search across PubMed, Scopus, Web of Science, and Google Scholar. Specific search strings incorporating keywords were structured using Boolean operators such as (“stapes prosthesis” OR “stapedial prosthesis” OR “stapes implant”) AND (“nitinol” OR “heat nitinol” OR “superelastic nitinol”) AND (“otosclerosis” OR “middle ear surgery”) to optimize the relevance of search results. The search was limited to articles published up to December 2023 and restricted to English-language studies.

**Figure 1:**
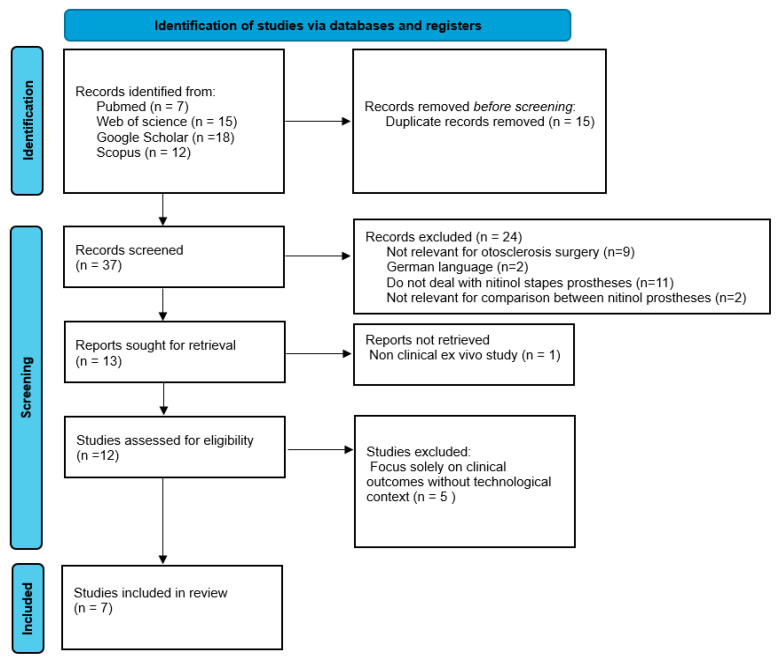
PRISMA diagram.

The systematic review adhered to the Preferred Reporting Items for Systematic Reviews and Meta-Analyses (PRISMA) guidelines to ensure transparency and methodological rigor (Figure 1).

Studies were included if they reported postoperative air–bone gap (ABG) thresholds, specifically ABG ≤ 10 dB or between 10 and 20 dB; provided data on audiological outcomes, surgical success rates, defined as achieving an ABG of ≤10 dB (optimal success) or between 10 and 20 dB (acceptable success), and complication profiles; and discussed the technological evolution or design differences between heat-activated and superelastic nitinol stapes prostheses. Exclusion criteria included studies unrelated to otosclerosis surgery; articles published in languages other than English or without available translations; studies without a specific focus on nitinol stapes prostheses; studies lacking comparative data on heat-activated and superelastic designs; and ex vivo or experimental studies.

The initial search yielded 37 articles. After removing duplicates, 24 studies were excluded during the title and abstract screening for the following reasons: nine were unrelated to otosclerosis surgery, two were published in German without available translations, eleven did not specifically address nitinol stapes prostheses, and two lacked comparative data on heat-activated and superelastic designs.

Thirteen articles underwent full-text review. One ex vivo study and five articles focusing solely on audiological or clinical outcomes, without addressing technological or design aspects of nitinol prostheses, were excluded. Ultimately, seven studies met all the inclusion criteria and were included in the review.

Data extraction focused on key parameters, including postoperative ABG thresholds, surgical success rates (defined as ABG ≤ 10 dB), complication rates, patient demographics, and prosthesis characteristics (e.g., type, material, design). Two reviewers independently performed data extraction using a standardized form. Discrepancies were resolved through discussion or consultation with a third reviewer.

The quality of included studies was evaluated using the Methodological Index for Non-Randomized Studies (MINORS), which assesses aspects such as endpoint clarity, follow-up appropriateness, and data collection methods. Scores for each study were tabulated, with higher scores reflecting greater methodological reliability. Publication bias was assessed qualitatively. Due to the limited number of studies included, statistical tests for funnel plot asymmetry, such as Egger’s test, were not performed. This limitation is discussed in the relevant section.

Statistical analyses were conducted to evaluate audiological outcomes and complication rates between heat-activated and superelastic prostheses. A random-effects model was employed for the meta-analysis to account for inter-study variability, estimating the pooled mean air–bone gap (ABG) reduction and its 95% confidence intervals (CI). Heterogeneity among studies was assessed using the $I^{2}$ statistic and *p*-value for Cochran’s Q test. Comparative analyses were performed to evaluate surgical success, defined as ABG ≤ 10 dB, and complication rates. Proportions of successful outcomes were compared using chi-square tests or Fisher’s exact tests, as appropriate. Odds ratios (ORs) with 95% CIs were calculated to determine the relative likelihood of surgical success and complication rates between the two prosthesis types. For studies reporting longitudinal outcomes, descriptive statistics were used to summarize ABG changes across follow-up intervals. Kaplan–Meier analysis was considered for assessing the stability of hearing outcomes over time, though data limitations prevented its full implementation.

## 3. Results

A total of seven studies were included in this review (Table 1), four of which focused on heat-activated nitinol prostheses and three on superelastic nitinol prostheses. The studies, published between 2011 and 2019, collectively analyzed data from 273 patients: 149 received heat-activated prostheses and 124 received superelastic prostheses. Most of the included studies employed retrospective cohort designs, while case series were used to address complex anatomical cases and revision surgeries (Table 1).

### 3.1. Summary of Quality Assessment

The quality assessment, using the Methodological Index for Non-Randomized Studies (MINORS), of the seven included studies reveals that the majority demonstrate good methodological quality, with most of them being retrospective studies. Studies such as Gerlinger et al. (2014) [7], Heywood et al. (2019) [10] and Hornung et al. (2011) [9] received high scores, showing clearly defined objectives, well-structured inclusion criteria and adequate follow-up periods. These studies provided robust clinical data on the effectiveness of nitinol stapes prostheses.

However, a few studies, such as Iannella et al. (2018) [11] and Teschner et al. (2019) [12], were rated as having moderate quality due to shorter follow-up periods or due to their designs being case series. These limitations may impact the generalizability of their findings but can still provide useful insights into the performance of nitinol prostheses.

Overall, the quality of evidence across these studies is considered high, although certain methodological limitations, particularly in terms of follow-up duration and study design, should be considered when interpreting the results.

### 3.2. Audiological Outcomes

**Figure 2:**
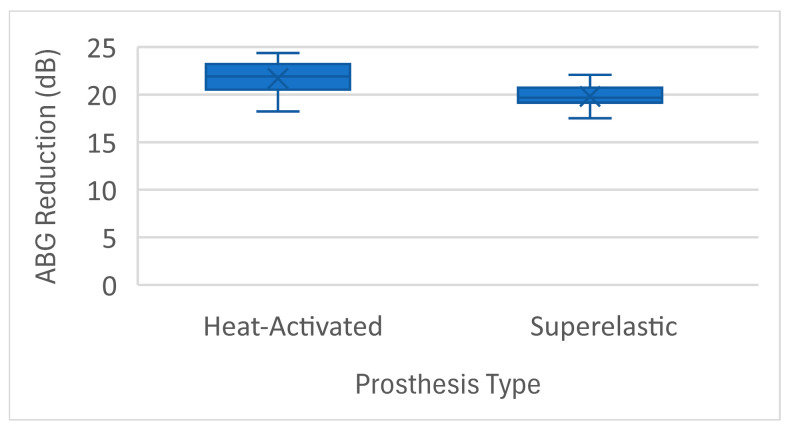
Distribution of ABG reduction: heat-activated vs. superelastic.

The audiological performance of heat-activated and superelastic nitinol prostheses was assessed through air–bone gap (ABG) reduction and success rates. Across the seven studies included, heat-activated prostheses achieved a mean ABG reduction of 22.2 dB (±6.45 dB) (Table 2), while superelastic prostheses achieved 19.9 dB (±6.34 dB) (Table 3). The Shapiro–Wilk test confirmed that ABG reduction data were normally distributed for both groups (Figure 2).

**Figure 3:**
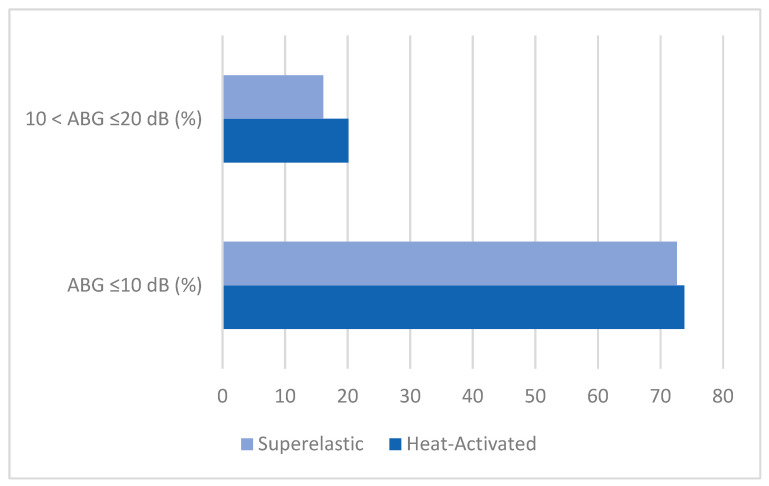
Comparison of success rates between heat-activated and superelastic prostheses: no statistically significant differences were observed (*p* > 0.05). Both prosthesis types demonstrate comparable success rates for both optimal success (ABG ≤ 10 dB) and acceptable success (ABG between 10 and 20 dB).

Surgical success rates, defined as achieving an ABG of ≤10 dB (optimal success) or between 10 and 20 dB (acceptable success), were analyzed in 273 patients (Table 4). The analysis of audiological outcomes revealed that the proportion of patients achieving an air–bone gap (ABG) ≤ 10 dB was comparable between heat-activated and superelastic prostheses. Among studies reporting outcomes for heat-activated prostheses, the percentage of patients with ABG ≤ 10 dB ranged from 64.5% to 83.3%, with Gerlinger et al. [7] reporting the highest success rate (83.3%). For superelastic prostheses, the success rates ranged from 32.3% to 83.3%, with Iannella et al. [11] reporting the highest proportion (83.3%). Similarly, the percentage of patients with 10 < ABG ≤ 20 dB was generally low across both groups, ranging from 10.4% to 48.4% for heat-activated prostheses and 10.0% to 17.9% for superelastic prostheses. Notably, Hornung et al. [9] (heat-activated group) reported the highest rate of moderate outcomes (48.4%). The chi-squared test revealed no statistically significant difference in the success rates for ABG ≤ 10 dB between heat-activated and superelastic prostheses (χ^2^ = 0.597, *p* > 0.05). Furthermore, the odds ratio (OR) for achieving ABG ≤ 10 dB with heat-activated prostheses compared to superelastic prostheses was 0.82 (95% CI: 0.43–1.54), indicating no significant association (Figure 3). These findings suggest that both prosthesis types achieve similar high audiological success rates (ABG ≤ 10 dB), with no significant difference in their performance across studies.

### 3.3. Complications

Complications were analyzed separately for each type of prosthesis, focusing on specific issues (Table 5).

**Figure 4:**
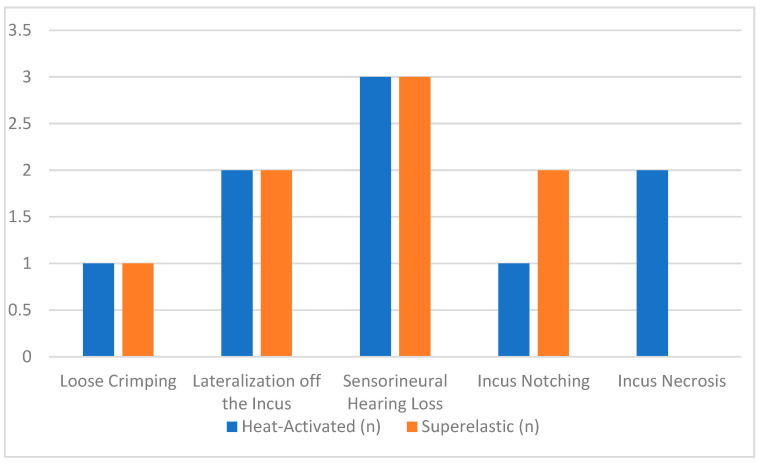
Complication rates by prosthesis type.

The analysis of complications showed a similar overall incidence between heat-activated and superelastic prostheses, with 6.0% (9/149) for heat-activated and 5.6% (7/124) for superelastic prostheses. The most reported complications included sensorineural hearing loss (2.2%), lateralization off the incus (1.5%), incus notching (1.1%), loose crimping (0.7%) and incus necrosis (0.7%). Among heat-activated prostheses, sensorineural hearing loss and incus necrosis were the most frequently reported complications (2.0% and 1.3%, respectively). For superelastic prostheses, lateralization off the incus and sensorineural hearing loss were the most common issues (1.6% and 2.4%, respectively). Statistical analysis revealed no significant difference in the overall complication rates between the two groups (*p* = 0.79) (Figure 4). The odds ratio (OR) for heat-activated versus superelastic prostheses was 1.07 (95% CI: 0.33–3.19), indicating no significant association. These findings suggest that both prosthesis types are associated with a low and comparable risk of complications.

### 3.4. Temporal Analysis of Follow-Up

The analysis of follow-up data highlights both short-term and long-term performance of heat-activated and superelastic nitinol prostheses. Stability and audiological outcomes were evaluated over follow-up periods ranging from 3 to 114 months.

Heat-activated prostheses achieved stable air–bone gap (ABG) reductions in 70.1% to 97% of cases across follow-up periods, with most patients maintaining improvements at 12 months and beyond.

Superelastic prostheses demonstrated high stability over time, with ABG ≤ 10 dB achieved in 62% to 97% of cases.

### 3.5. Meta-Analysis

**Figure 5:**
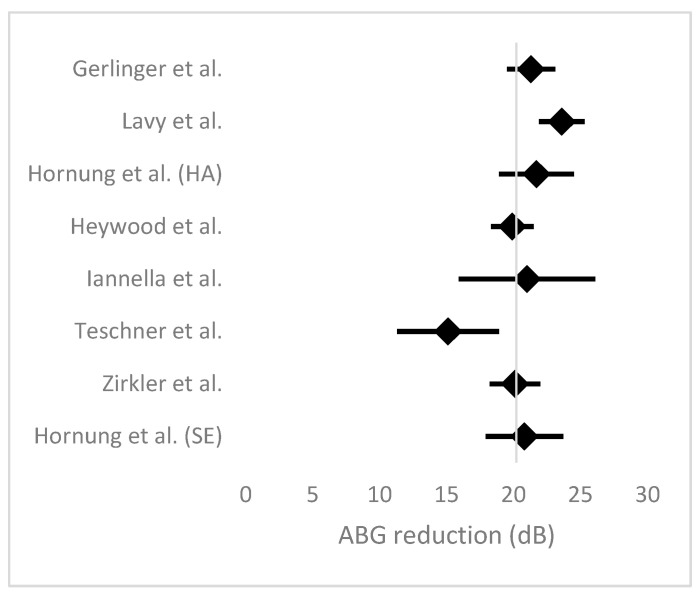
Forest plot illustrating the mean air–bone gap (ABG) reduction (in decibels) across the included studies. Each study is represented by a horizontal line, indicating the 95% confidence interval (CI) for the mean ABG reduction, with a central dot representing the study’s mean. The vertical dashed line represents the overall pooled mean ABG reduction, calculated using a random-effects model. The pooled 95% CI is shaded around the mean. Studies with narrower CIs reflect greater precision due to larger sample sizes or lower variance [7,8,9,10,11,12,13].

A meta-analysis was performed to evaluate the mean air–bone gap (ABG) reduction following stapes surgery using heat-activated and superelastic prostheses across seven studies. A random-effects model was employed to account for heterogeneity among studies. The pooled mean ABG reduction was estimated at 20.2 dB (95% CI: 19.47–20.95 dB), indicating a significant and clinically meaningful improvement in hearing outcomes. Individual study estimates ranged from 15.1 dB to 23.6 dB, with narrower confidence intervals observed in studies with larger sample sizes. The analysis revealed moderate heterogeneity, suggesting some variability in outcomes across studies, potentially attributable to differences in surgical techniques, patient populations, or prosthesis designs. A forest plot (Figure 5) visualized the pooled mean and the confidence intervals for each study, demonstrating the consistency of the results. These findings support the efficacy of both prosthesis types in achieving substantial ABG reduction after stapes surgery.

## 4. Discussion

This systematic review and meta-analysis provide a comprehensive evaluation of the audiological outcomes, complications, and comparative efficacy of heat-activated and superelastic prostheses in stapes surgery. The pooled mean air–bone gap (ABG) reduction of 20.2 dB (95% CI: 19.47–20.95 dB) reflects a substantial improvement in conductive hearing loss, consistent across the included studies. For example, Lavy et al. [8] reported the highest mean ABG reduction of 23.6 dB, highlighting the effectiveness of heat-activated prostheses, while Teschner et al. [12] observed a lower reduction of 15.1 dB, which may reflect differences in surgical technique or patient selection criteria, as suggested by the analyses presented in the original studies. These results support the efficacy of both types of prostheses in achieving meaningful audiological improvements.

The analysis of complications revealed distinct patterns between the two types of prostheses, with both showing low overall rates. For heat-activated prostheses, complications were reported in 6.0% of cases. Neurosensorial hearing loss was the most frequently noted complication, occurring in 2.0% of patients. For instance, Hornung et al. (2011) identified two cases of post-operative neurosensorial hearing loss with heat-activated prostheses, attributing these to potential inner ear trauma during surgery [9]. Similarly, Lavy et al. (2014) [8] reported a single case of sensorineural hearing loss, reinforcing the importance of delicate surgical techniques.

Incus necrosis was another complication observed with heat-activated prostheses, affecting 1.3% of patients. Gerlinger et al. (2014) and Lavy et al. (2014) documented a case where prolonged pressure from the prosthesis likely disrupted blood flow to the incus, leading to necrosis [7,8]. This underscores the need for careful prosthesis crimping and positioning during surgery. Superelastic prostheses in contrast showed a lower risk of incus necrosis due to the distributed pressure across the incus [12].

In the other hand, the complication rate for superelastic prostheses was slightly lower at 5.6%. Lateralization off the incus emerged as the most notable issue in studies such as Heywood et al. (2019) and Hornung et al. (2011), where this complication affected 1.6% of patients. This problem is often linked to post-operative prosthesis displacement, emphasizing the importance of secure initial placement [9,10].

Other complications included loose crimping, which was reported in Lavy et al. (2014) [8] and in Iannella et al. (2018) [11], affecting 16.7% of their small cohort of patients undergoing revision surgery. This finding highlights the challenge of achieving secure fixation in revision cases. Incus notching, a rare but significant issue, was reported in 1.1% of cases, with Zirkler et al. (2016) noting this complication in one patient. This problem typically arises from prolonged mechanical pressure exerted by the prosthesis [13].

Despite these complications, the overall safety of both prosthesis types is supported by studies like Heywood et al. (2019) [10] and Gerlinger et al. (2014) [7], where complication rates remained low and outcomes favorable. The adaptability of superelastic prostheses, as highlighted by Teschner et al. (2019) [12] and Zirkler et al. (2016) [13], may reduce procedural complexity and complications such as crimping errors. Conversely, heat-activated prostheses, demonstrated by Lavy et al. (2014) and Hornung et al. (2011), offer reliable fixation, particularly in primary surgeries [8,9].

The temporal analysis of follow-up data confirmed the long-term stability of both prosthesis types, with success rates ranging from 62% to 97% for superelastic prostheses and 70.1% to 97% for heat-activated prostheses over follow-up periods of 3 to 114 months. However, late-stage complications, such as incus necrosis, were more frequently observed with heat-activated prostheses, likely due to excessive pressure during crimping caused by the variability reliance on thermal activation [9,10]. Superelastic prostheses, by contrast, demonstrated greater long-term predictability, particularly in dynamic middle ear environments, owing to their adaptive and flexible design [11].

While both prostheses provided stable outcomes, superelastic prostheses demonstrated more predictable long-term performance and fewer late-stage failures, making them particularly advantageous in revision surgeries (Teschner et al., 2019) [12].

The evolution of nitinol stapes prostheses has introduced innovative designs that address specific surgical needs. Heat-activated prostheses, such as those by Kurz and Grace Medical, incorporate auto-crimping mechanisms that simplify surgical procedures but are associated with higher complication rates. Conversely, superelastic prostheses, including models from Audio Technologies and Olympus, prioritize adaptability and safety through features like self-crimping mechanisms and customizable sizes [6,7].

In summary, while both prosthesis types are effective in restoring hearing, superelastic nitinol prostheses offer a superior safety profile and more predictable outcomes. These findings emphasize the critical role of prosthesis design and manufacturing in determining clinical success.

### 4.1. Limitations

This review has several limitations that should be considered when interpreting the results. First, the included studies were predominantly retrospective, with inherent risks of bias and variability in data quality. Second, the heterogeneity observed in the meta-analysis ($I^{2}$ = 46%) may reflect differences in surgical techniques, patient populations, and follow-up durations. Additionally, the surgeon’s experience plays a fundamental role in the outcomes of stapes surgery. The annual number of procedures performed by the authors of the included studies varies significantly, potentially impacting the results. For instance, experienced surgeons who have performed a high number of procedures may achieve greater precision in prosthesis placement, thereby reducing complications such as prosthesis displacement or incus necrosis. However, many of the included studies did not report these data, representing a critical gap to address when standardizing surgical outcomes. Third, the absence of randomized controlled trials limits the ability to draw definitive conclusions about the superiority of one prosthesis type over the other. Finally, the lack of a publication bias assessment, such as a funnel plot or Egger’s test, represents a methodological gap.

### 4.2. Implications and Future Directions

The findings of this review emphasize the importance of individualized prosthesis selection based on patient-specific factors and surgical expertise. Both heat-activated and superelastic nitinol prostheses are associated with high audiological success rates and low complication profiles, making them suitable for most otosclerosis cases. However, future research should focus on conducting randomized controlled trials to provide high-quality evidence, on standardizing outcome measures to facilitate comparisons across studies, exploring long-term stability of audiological outcomes beyond 5 years and investigating patient-reported outcomes, such as quality of life and hearing satisfaction, to complement objective measures. While this review focuses primarily on audiological outcomes and complication rates, cost analysis is a critical factor for decision-making in clinical and policy settings. This review does not include cost-effectiveness analyses due to the lack of available data in the included studies. Heat-activated prostheses may offer short-term cost advantages due to their simpler surgical application and reduced operative time, particularly in primary surgeries. Conversely, the long-term stability and lower complication rates of superelastic prostheses may translate into cost savings by reducing the need for revisions or additional procedures. Future studies should evaluate the economic implications of these prostheses to provide a more comprehensive perspective on their clinical utility.

## 5. Conclusions

This systematic review and meta-analysis underline the comparable audiometric performance of heat-activated and superelastic nitinol stapes prostheses in otosclerosis surgery, with both prosthesis types achieving significant reductions in the air–bone gap (ABG). Both heat-activated and superelastic demonstrate low complication rates, with differences in the types of complications observed. Surgical technique, patient-specific factors, and prosthesis design all play critical roles in minimizing complications and optimizing outcomes. Despite their equivalence, the self-crimping mechanism of superelastic prostheses minimizes reliance on surgical precision, reduces mechanical failures, and enhances long-term stability. In contrast, heat-activated prostheses are associated with slightly higher complication rates, which are linked to the technical demands of thermal activation and manual placement [7,12]. In conclusion, the choice between heat-activated and superelastic prostheses should consider both the biomechanical properties of the materials and the surgeon’s level of experience. Superelastic prostheses, with their adaptive design, offer advantages in safety and long-term predictability, especially in the hands of experienced surgeons. Conversely, heat-activated prostheses may be preferred for their ease of use in primary surgical contexts. These findings highlight the importance of selecting prosthesis technologies not only for their audiometric outcomes but also for their safety profiles and reliability.

Future research should focus on long-term follow-up studies with larger patient cohorts to validate these findings further and explore the influence of emerging technological innovations on surgical outcomes. Additionally, studies examining cost-effectiveness and patient-reported outcomes could provide critical insights for optimizing prosthesis selection and improving decision-making in otosclerosis surgery. Such research could enhance the clinical utility of these devices, ensuring better alignment with patient needs and health system priorities.

## Figures and Tables

**Table 1:** Characteristics of the studies included in the systematic review. The table summarizes the year of publication, study design, sample size and type of prosthesis analyzed (heat-activated or superelastic nitinol. A total of 273 patients were examined across seven studies, with 149 treated using heat-activated prostheses and 124 using superelastic prostheses.

Study	Year	Prosthesis Type	Design	Sample Size	Heat-Activated	Superelastic
Gerlinger et al. [7]	2014	Heat-activated	Retrospective cohort	48	48	-
Heywood et al. [10]	2019	Superelastic	Retrospective cohort	56	-	56
Iannella et al. [11]	2018	Superelastic	Case series	6	-	6
Teschner et al. [12]	2019	Superelastic	Retrospective cohort	10	-	10
Lavy et al. [8]	2014	Heat-activated	Retrospective cohort	70	70	-
Zirkler et al. [13]	2016	Superelastic	Retrospective cohort	21	-	21
Hornung et al. [9]	2011	Both	Retrospective cohort	62	31	31
Total	-	-	-	273	149	124

**Table 2:** Mean of ABG reduction and standard deviation results in heat-activated group.

Heat-Activated Studies	ABG Reduction (dB)	Standard Deviation (dB)
Gerlinger et al. [7]	21.3	3.6
Lavy et al. [8]	23.6	7.3
Hornung et al. [9]	21.7	7.9
Mean	22.2	±6.45

**Table 3:** Mean of ABG reduction and standard deviation results in the superelastic group.

Superelastic Studies	ABG Reduction (dB)	Standard Deviation (dB)
Heywood et al. [10]	19.8	6.1
Iannella et al. [11]	21.0	6.2
Teschner et al. [12]	15.1	6.1
Zirkler et al. [13]	19.5	4.3
Hornung et al. [9]	20.8	8.3
Mean	19.9	±6.34

**Table 4:** Results in surgical success rates, defined as achieving an ABG of ≤10 dB (optimal success) or between 10 and 20 dB (acceptable success).

Study	Prosthesis	ABG ≤ 10 dB (n/N (%))	10 ≤ ABG ≤ 20 dB (n/N (%))
Gerlinger et al. [7]	Heat-Activated	40/48 (83.3%)	5/48 (10.4%)
Lavy et al. [8]	Heat-Activated	50/70 (71.4%)	10/70 (14.3%)
Hornung et al. [9]	Heat-Activated	20/31 (64.5%)	15/31 (48.4%)
Heywood et al. [10]	Superelastic	42/56 (77.8%)	6/56 (17.9%)
Iannella et al. [11]	Superelastic	5/6 (83.3%)	1/6 (16.7%)
Teschner et al. [12]	Superelastic	7/10 (70.0%)	1/10 (10.0%)
Zirkler et al. [13]	Superelastic	15/21 (71.4%)	3/21 (14.3%)
Hornung et al. [9]	Superelastic	10/31 (32.3%)	5/31 (16.1%)

**Table 5:** Summary of complications reported across the studies.

Study	Prosthesis Type	Loose Crimping *(n*, %)	Lateralization off the Incus (*n*, %)	Sensorineural Hearing Loss (*n*, %)	Incus Notching (*n*, %)	Incus Necrosis (*n*, %)	Total Complications (*n*, %)	Total Patients (*N*)
Gerlinger et al. [7]	Heat-Activated	0 (0.0%)	0 (0.0%)	0 (0.0%)	1 (2.1%)	1 (2.1%)	2 (4.2%)	48
Lavy et al. [8]	Heat-Activated	1 (1.4%)	1 (1.4%)	1 (1.4%)	0 (0.0%)	1 (1.4%)	4 (5.7%)	70
Hornung et al. [9]	Heat-Activated	0 (0.0%)	1 (3.2%)	2 (6.5%)	0 (0.0%)	0 (0.0%)	3 (9.7%)	31
Heywood et al. [10]	Superelastic	0 (0.0%)	1 (1.8%)	0 (0.0%)	1 (1.8%)	0 (0.0%)	3 (5.4%)	56
Iannella et al. [11]	Superelastic	1 (16.7%)	0 (0.0%)	1 (16.7%)	0 (0.0%)	0 (0.0%)	2 (33.3%)	6
Teschner et al. [12]	Superelastic	0 (0.0%)	0 (0.0%)	0 (0.0%)	0 (0.0%)	0 (0.0%)	0 (0.0%)	10
Zirkler et al. [13]	Superelastic	0 (0.0%)	0 (0.0%)	1 (4.8%)	1 (4.8%)	0 (0.0%)	2 (9.5%)	21
Hornung et al. [9]	Superelastic	0 (0.0%)	1 (3.2%)	1 (3.2%)	0 (0.0%)	0 (0.0%)	2 (6.5%)	31
Totals	-	2 (0.7%)	4 (1.5%)	6 (2.2%)	3 (1.1%)	2 (0.7%)	17 (6.2%)	273
